# Morbus Castleman in der rheumatologischen Praxis

**DOI:** 10.1007/s00393-023-01393-8

**Published:** 2023-08-25

**Authors:** M. Schmalzing, O. Sander, M. Seidl, R. Marks, N. Blank, I. Kötter, M. Tiemann, M. Backhaus, B. Manger, K. Hübel, U. Müller-Ladner, J. Henes

**Affiliations:** 1 Medizinische Klinik und Poliklinik II, Universitätsklinikum Würzburg, Oberdürrbacher Str. 6, 97080 Würzburg, Deutschland; 2 Klinik für Rheumatologie, Universitätsklinikum Düsseldorf, Düsseldorf, Deutschland; 3 Institut für Pathologie, Universitätsklinikum Düsseldorf, Düsseldorf, Deutschland; 4 Klinik für Innere Medizin I, Universitätsklinikum Freiburg, Freiburg, Deutschland; 5 Universitätsklinikum Heidelberg, Heidelberg, Deutschland; 6 Klinik für Rheumatologie und Immunologie, Klinikum Bad Bramstedt, Bad Bramstedt, Deutschland; 7 Rheumatologie, Universitätsklinikum Hamburg-Eppendorf, Hamburg, Deutschland; 8 Institut für HämatoPathologie Hamburg, Hamburg, Deutschland; 9 Abt. Innere Medizin – Rheumatologie und klinische Immunologie, Park-Klinik Weissensee (Berlin), Berlin, Deutschland; 10 Medizinische Klinik 3 – Rheumatologie und Immunologie, Universitätsklinikum Erlangen, Erlangen, Deutschland; 11 Klinik I für Innere Medizin, Universitätsklinikum Köln, Köln, Deutschland; 12 Abteilung für Rheumatologie und Klinische Immunologie, Kerckhoff Klinik Bad Nauheim, Bad Nauheim, Deutschland; 13 Medizinische Universitätsklinik Abt. II, Universitätsklinikum Tübingen, Tübingen, Deutschland

**Keywords:** Morbus Castleman, Idiopathischer MCD, Lymphknotenhyperplasie, Histopathologie, IL‑6, Castleman’s disease, Idiopathic MCD, Lymph node hyperplasia, Histopathology, IL‑6

## Abstract

Der Begriff „Morbus Castleman“ umfasst eine Gruppe von seltenen lymphoproliferativen Krankheitsbildern, die histopathologische Gemeinsamkeiten in der Lymphknotenbiopsie aufweisen. Erst seit wenigen Jahren stehen diagnostische Kriterien sowie ein spezifischer ICD-10-Code zur Verfügung. Mit den eingangs aufgeführten Fallbeispielen wird veranschaulicht, dass es für die Diagnosestellung einer engen Zusammenarbeit zwischen Klinikern und Pathologen bedarf. Für eine optimale histopathologische Beurteilung ist der Pathologe zudem auf die Entnahme eines vollständigen Lymphknotens angewiesen. Vor der hinsichtlich der Prognose und Therapie bedeutsamen Abgrenzung eines potenziell fatal verlaufenden, multilokulären idiopathischen Morbus Castleman von der resezierbaren, lokalisierten Form setzt die frühzeitige Diagnose aber voraus, dass das Krankheitsbild differenzialdiagnostisch überhaupt erst in Betracht gezogen wird. Verschiedene Immunphänomene und Überlappungen mit u. a. autoimmun bedingten Erkrankungen können die Wahrscheinlichkeit von Fehldiagnosen oder unerkannten Fällen auch im klinischen Alltag des Rheumatologen erhöhen. Intention der vorliegenden Übersicht war es daher, auch auf die Ähnlichkeiten mit differenzialdiagnostisch relevanten, Autoimmunerkrankungen hinzuweisen und Situationen aufzuzeigen, die eine Überprüfung der bisherigen Diagnose rechtfertigen.

## Abstract (en)

The term Castleman’s disease encompasses a group of rare lymphoproliferative diseases that show histopathological similarities in lymph node biopsy. Diagnostic criteria and a specific ICD-10 code have been available for a few years. Case studies listed at the beginning illustrate that close cooperation between clinicians and pathologists is required to enable a reliable diagnosis. For an optimal histopathological assessment, the pathologist is also dependent on the removal of a complete lymph node. Before distinguishing a potentially fatal multicentric idiopathic Castleman’s disease from the resectable unicentric form, which is important in terms of prognosis and treatment, early diagnosis presupposes that Castleman’s disease is considered in the differential diagnosis. Various immune phenomena and overlaps with autoimmune diseases can increase the probability of misdiagnosis or undetected cases in the clinical routine of rheumatologists. The intention of the present overview is therefore to point out the similarities with autoimmune diseases that are relevant for differential diagnoses and to point out situations that justify a review of the previous diagnosis.

Patienten mit einer „Castleman-Krankheit“ („Castleman disease“ [CD]) dürften zu einem maßgeblichen Anteil aufgrund der heterogenen Klinik, Verläufe und Überlappungen mit malignen, infektiös oder autoimmun bedingten Erkrankungen sowie Syndromen fehldiagnostiziert bzw. unerkannt sein. Insbesondere der idiopathische multizentrische CD (iMCD) stellt als potenziell lebensbedrohliche Systemerkrankung eine wichtige Differenzialdiagnose für die rheumatologische Praxis dar.

Die Erstbeschreibung des Morbus Castleman geht auf den amerikanischen Pathologen Benjamin Castleman zurück, der das Krankheitsbild erstmals 1954 bei Patienten mit solitären mediastinalen Lymphknotenhyperplasien als „eigenartige Form der Lymphknotenvergrößerung“ beschrieb [1]. Nachfolgende Fallbeispiele verdeutlichen, warum die differenzialdiagnostische Abgrenzung zu Autoimmunerkrankungen des rheumatologischen Formenkreises oftmals besonders herausfordernd ist.

## Kasuistik 1

**Figure 1:**
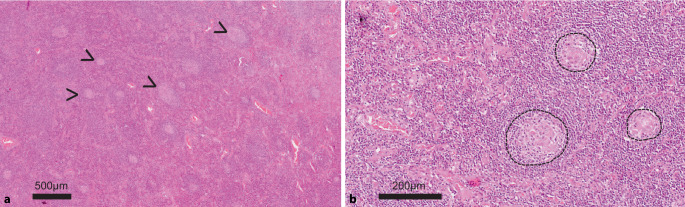

Eine 62-jährige Patientin wurde mit Verdacht auf Kollagenose erstmals einer universitären Rheumatologie zugewiesen. Die Patientin stellte sich mit einem stammbetonten Erythem und einer schon länger bestehenden, zuletzt regredienten, Lymphadenopathie vor. Weiterhin wies die Patientin ein anamnestisch vorbekanntes Raynaud-Syndrom, eine Lungenfibrose sowie eine pulmonalarterielle Hypertonie (PAH) der WHO-Funktionsklasse II auf. Im aktuellen Laborbefund stand eine ausgeprägte polyklonale Gammopathie im Vordergrund (bei Ausschluss einer Amyloidose). Serologisch ergaben sich zudem hochtitrige ANA-Werte (1:20.480, gesprenkeltes Muster) sowie ein positiver Antikörperbefund gegen RNP, Sm, SSA, SSB und dsDNS. Retrospektiv wurden 8 Jahre zuvor erhöhtes Gesamteiweiß im Serum (9,4 g/dl) und eine milde Anämie (10,3 g/dl) berichtet, mehr als 1 Jahr zuvor eine ausgeprägte polyklonale Hypergammaglobulinämie (IgG > 5 g/dl). Das C‑reaktive Protein (CRP) war im Verlauf entweder normwertig oder auf bis zu 11 mg/dl erhöht. Aus der Vorgeschichte war ferner bekannt, dass die Patientin bereits mehrfach wegen Episoden mit Fieber, erhöhten Entzündungswerten, Exanthemen und weiteren Immunphänomenen stationär behandelt wurde. In der Regel wurde zunächst ein Infekt behandelt, wobei es unter Antibiose zu einer Besserung der Symptomatik gekommen sein soll. Relevante therapeutische Effekte unter Steroiden wurden nur unter sehr hohen Dosierungen beobachtet. Im Rahmen einer Myositisabklärung vor 6 Monaten wurde eine Lymphknotenbiopsie axillär links durchgeführt, zunächst befundet als „unspezifische Lymphadenitis“. Einen Monat darauf erfolgte eine nachträgliche Aufarbeitung durch ein Lymphom-Referenzzentrum: Demnach korrespondierte der histopathologische Befund mit einem MCD mit sekundär-lymphofollikulärer Hyperplasie (Abb. 1).

## Kasuistik 2

**Figure 2:**
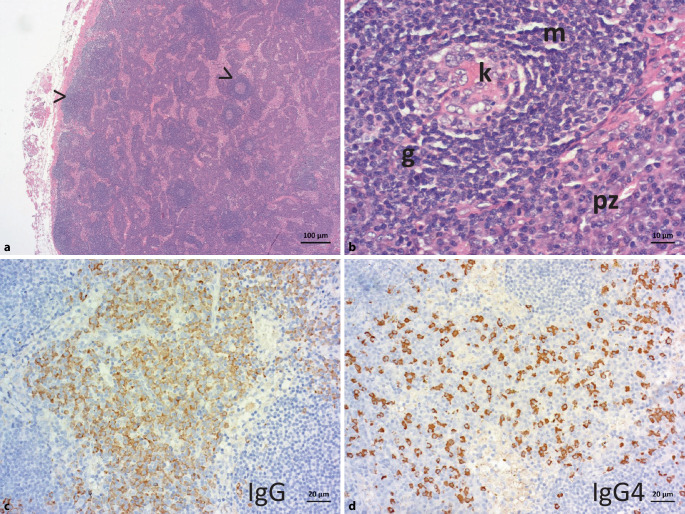

Ein 22-jähriger Patient stellte sich mit Thoraxschmerzen und unklarer Infektkonstellation in der Notaufnahme vor. Die Initialdiagnostik ergab Hinweise auf ein akutes Nierenversagen, eine respiratorische Partialinsuffizienz und eine multilokuläre Lymphadenopathie (hilär, mesenterial, retroperitoneal und parailiakal) sowie eine Thymushyperplasie. Eine mehrfach wiederholte Erregerdiagnostik blieb im Verlauf negativ. Im Zuge des innerhalb von 3 Wochen verschlechterten Allgemeinzustands mit beidseitigen Pleuraergüssen und Aszites erfolgte die Übernahme auf die Intensivstation. Histologisch bestanden eine chronisch fibrosierende Mediastinitis und granulierende Pleuritis, markmorphologisch eine trilineär gesteigerte Hämatopoese mit führend vermehrt Megakaryozyten. Im Zuge einer erneuten Intervention bei mediastinalen Lymphknotenpaketen erfolgte die Asservation eines vollständigen Lymphknotens. In der histopathologischen Untersuchung ergab sich eine MCD-typische Histologie (Abb. 2).

## Systematik

**Figure 3:**
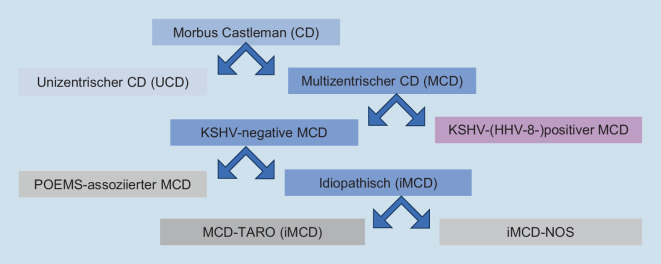

Aufgrund der klinischen Präsentation wird ein multizentrischer vom unizentrischen Morbus Castleman abgegrenzt. Beim MCD („multicentric Castleman disease“ [MCD]) handelt es sich im Unterschied zur unizentrischen Castleman-Krankheit („unicentric Castleman disease“ [UCD]) nicht um eine lokalisierte und gut behandelbare Hyperplasie des lymphatischen Gewebes: Die multilokuläre Lymphadenopathie beim MCD kann mit einer schwerwiegenden, systemisch progredienten Erkrankung assoziiert sein und unbehandelt letal verlaufen [2]. Ätiopathogenetisch bedeutsam ist die weitere Unterteilung in einen mit HHV‑8 (humanes Herpesvirus 8; auch: Kaposi-Sarkom-assoziiertes Herpesvirus [KSHV]) assoziierten bzw. HHV-8-negativen MCD unbekannter Ätiologie (idiopathischer MCD [iMCD]) [3]. Die HHV8-assoziierte MCD wird am häufigsten bei HIV-infizierten bzw. immunkompromittierten Personen beobachtet [2, 3]. Beim erstmals in Japan beschriebenen Subtyp iMCD-TAFRO steht das unlängst etablierte Akronym „TAFRO“ für Thrombozytopenie, Aszites, Fieber, retikuläre Fibrose im Knochenmark und Organomegalie [4]. Daneben weisen Patienten mit „nicht anderweitig spezifizierbarem“ iMCD (iMCD-NOS) kein TAFRO-Syndrom auf [5]. Selten ist der iMCD mit einem POEMS-Syndrom aus Polyneuropathie, Organomegalie, Endokrinopathie, monoklonaler Gammopathie und Hautveränderungen vergesellschaftet (POEMS-assoziierter iMCD) (Abb. 3).

## Epidemiologie

Durch die Seltenheit der Erkrankung ist die Datenlage in Bezug auf die Häufigkeit des Morbus Castleman unbefriedigend. Die Einschränkungen bei der Evaluierbarkeit des Krankheitsbildes lassen sich auch auf die erst vor wenigen Jahren eingeführten diagnostischen Kriterien sowie spezifischen ICD-10-Code zurückführen (2016, 2017) [2, 5]. Sowohl in seiner Inzidenz als auch Prävalenz dürfte der Morbus Castleman aber unterschätzt sein [6]. Einer erstmals nach den neueren Diagnosekriterien vorgenommenen Auswertung von US-Krankenversicherungsdaten zufolge waren im Jahr 2017 etwa 3326 Patienten mit CD bzw. im Jahr 2018 ungefähr 5282 Patienten prävalent. Die auf das Jahr 2017 (2018) bezogene Inzidenz wurde mit 3,4 (3,1) bzw. Prävalenz mit 6,9 (9,7) iMCD-Fällen pro Million Individuen berechnet, wobei von einer untererfassten Prävalenz auszugehen ist. Grundsätzlich kann Morbus Castleman jedes Lebensalter betreffen. Das mediane Alter beim MCD liegt bei Erstdiagnose im 5. bis 6. Lebensjahrzehnt, beim UCD im 40. Lebensjahr. Männer scheinen beim MCD tendenziell häufiger betroffen zu sein als Frauen [7].

## Pathogenese

### UCD

Die Pathogenese des UCD ist erst unvollständig verstanden, wobei es sich jüngeren Erkenntnissen zufolge um einen neoplastischen Prozess der Lymphknotenstromazellen (LNSC) einbezüglich der follikulären dendritischen Retikulumzellen (FDC) handeln könnte [7, 8]. Bei 17 % der untersuchten UCD-Fälle wurden Mutationen im PDGFRB („platelet-derived growth factor receptor-β“) identifiziert [9]. Eine Assoziation mit Virusinfektionen wurde nicht nachgewiesen [5].

### MCD

Am bislang besten verstanden ist der Pathomechanismus beim HHV-8-positiven MCD (KSHV-MCD), welcher sowohl bei HIV-positiven als auch HIV-negativen, HHV-8-positiven Patienten beobachtet wird und zu einer polyklonalen Lymphoproliferation führt. Bis zu 30 % der B‑Lymphozyten in der Mantelzone der Lymphfollikel werden mit HHV‑8 infiziert, wobei fast ausschließlich B‑Zellen betroffen sind, die IgM-lambda-Leichtketten exprimieren. Virale Gene werden sowohl während der viralen Latenz (z. B. LANA) als auch während der lytischen Virusreplikation (z. B. virales Interleukin‑6 [vIL-6]) exprimiert und können im KSHV-MCD nachgewiesen werden. Als besonders charakteristisch gilt das virale Protein vIL‑6, das homolog zum humanen IL‑6 ist und v. a. in Plasmablasten beobachtet wird, die die Lymphfollikel umgeben. Neben der exzessiven Freisetzung von IL‑6 und vIL‑6 zeigen sich beim KSHV-MCD weitere inflammatorische Zytokine, wie z. B. IL-10, IL-1β und TNF, dysreguliert („Zytokinsturm“) [2].

Die Ätiologie des iMCD ist nicht endgültig geklärt, wobei mindestens einer der folgenden hypothetischen Pathomechanismen diskutiert wird: Autoimmunität, Autoinflammation, neoplastische Prozesse und/oder bislang unbekannte virale Infektionen von pathogenetischer Relevanz [3]. Es ist denkbar, dass unterschiedliche Pathomechanismen zum Zytokin- und Chemokinsturm führen bzw. einen ähnlichen klinischen Phänotyp verursachen. Dabei können neben IL‑6 weitere proinflammatorische Zytokine und Chemokine in die Pathogenese involviert sein, wie z. B. VEGF, IL‑1, IL‑8, CXCL13 oder TNF‑α [2, 3, 10]. Darüber hinaus könnten die T‑Zell-Aktivierung und Aktivierung der mTOR(„mammalian target of rapamycin“)- oder JAK-STAT3(„Janus kinase/signal transducer and activator of transcription 3“)- bzw. auch Typ-I-Interferon-Signalwege eine Rolle spielen. Im Zuge der (De‑)Aktivierung bestimmter Gene wird auch die Möglichkeit einer gesteigerten Inflammasomaktivität und damit autoinflammatorischen Genese diskutiert [2].

## Klinik

Die lokalisierte Form des *UCD* präsentiert sich in der Regel als sichtbare oder palpable Lymphadenopathie. Ein UCD kann symptomatisch werden, wenn benachbarte Strukturen verdrängt werden. Konstitutionelle Symptome wie Fatigue oder Gewichtsverlust sind beim UCD dagegen selten und für gewöhnlich nicht vorhanden [2]. Ein *MCD* verläuft dagegen selten asymptomatisch. Die Erkrankung zeigt einen schubweisen Verlauf, wobei die Patienten häufig im Schub schwerkrank sind. Eine oft ausgeprägte B‑Symptomatik mit Fieber, Nachtschweiß und Gewichtsverlust kann in der Zusammenschau mit Splenomegalie, sehr häufig auch Hepatomegalie, Anasarka (massive generalisierte Ödeme aufgrund von Hypalbuminämie) sowie unspezifisch erhöhten laborchemischen Entzündungsmarkern (v. a. BSG, CRP), Anämie und Hypergammaglobulinämie auf die Erkrankung hinweisen. Eine renale Beteiligung mit konsekutivem Nierenversagen und Proteinurie wird v. a. bei den HHV-8-negativen iMCD-Patienten beobachtet [11]. Bei einer Vielzahl von Krankheitsprozessen können Hypergammaglobulinämien das Resultat einer polyklonalen B‑Zell-Aktivierung sein. Bei der laborserologischen Befundinterpretation ist zu berücksichtigen, dass eine Hypergammaglobulinämie mit falsch positiven Immunserologien (z. B. Nachweis von mehreren Autoantikörpern) assoziiert sein kann [12]. Bei HHV-8-positiven MCD-Patienten ist die klinische Symptomatik tendenziell stärker ausgeprägt als bei Patienten mit iMCD, wie die Daten aus den beiden größten Fallserien bzw. Metaanalyse nahelegen (Tab. 1; [11, 13]). Renal lassen sich u. a. sekundäre Amyloidosen und membranoproliferative Glomerulonephritiden beobachten, pulmonal können die Patienten durch eine lymphoide interstitielle Pneumonitis (LIP), restriktive Lungenerkrankung oder Bronchiolitis obliterans auffällig werden. Als potenzielle Hautveränderungen sind u. a. Ausschlag, Hyperpigmentierung, tardives Hämangiom („Kirsch-Angiom“), paraneoplastischer Pemphigus und Kaposi-Sarkom zu berücksichtigen [7]. Weiterhin berichten viele Patienten über Fatigue, Inappetenz und Übelkeit [14]. In seltenen Einzelfällen wurden Patienten mit iMCD beschrieben, die RA(rheumatoide Arthritis)-ähnliche aktive Arthritiden entwickelten. Demnach könnte die T‑Zell-Aktivierung auch bei der Entstehung von Arthritiden eine pathophysiologisch relevante Rolle spielen und zum komorbiden Auftreten dieser RA-ähnlichen Symptomatik bei iMCD führen [15].

**Table 1:** 

	HHV-8-positiv (%)	HHV-8-negativ (%)
Fieber	88	73
Splenomegalie	78	41–78
Ödeme	40	23–78
Lungenbeteiligung	26	48
Hautauffälligkeiten	13	22
Nierenbeteiligung	6,5	41
Hämophagozytose	37	4

^a^Lungen- und Nierenbeteiligung traten häufig in Begleitung von Ödemen/Ergüssen auf

## Diagnostik und Histopathologie

**Figure 4:**
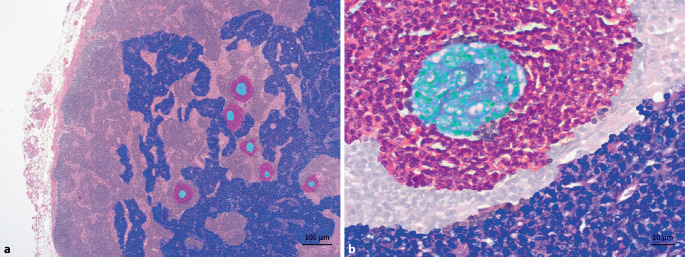

**Figure 5:**
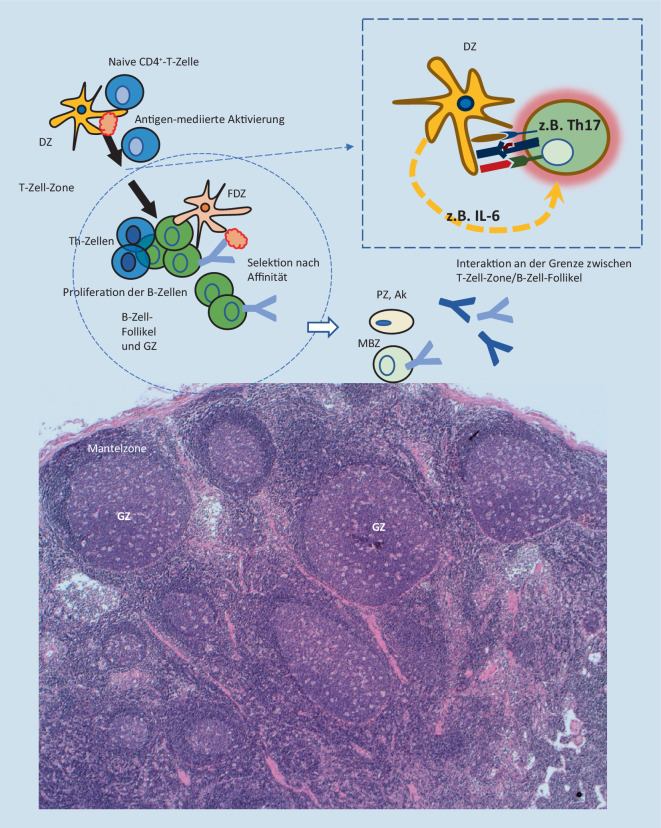

Am Beispiel des eingangs geschilderten Patientenfalls (Kasuistik 2) wird die CD-Histopathologie über die Falschfarbendarstellung nachvollziehbar: Der Patient wies regressive Keimzentren auf – mit deutlich vermindertem B‑Zell-Anteil bei kondensierten, Immunkomplex-beladenen follikulären dendritischen Zellen (Abb. 4). Demnach können beim CD regressive Keimzentren das Resultat einer deutlichen Plasmazellexpansion und überschießenden Antikörperproduktion sein, wodurch der Antigenzugang für Keimzentrums-B-Zellen zunehmend erschwert wird (Abb. 5).

**Figure 6:**
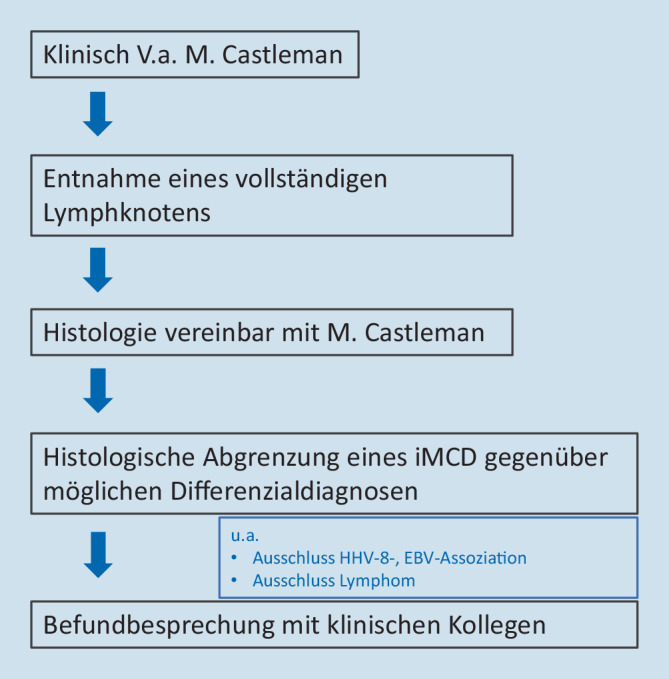

Aus Sicht des Pathologen sind 2 Voraussetzungen für eine frühzeitige Diagnose wesentlich: erstens, an die Differenzialdiagnose Morbus Castleman zu denken, und zweitens, die Entnahme eines vollständigen Lymphknotens zur optimalen histologischen Beurteilung. Eine Punktion ist, um die Diagnosestellung eines CD gesichert stellen zu können, nicht ausreichend. Die wesentlichen pathologischen Veränderungen umfassen eine follikuläre Hyperplasie mit regressiven, teils hyalin-vaskulär transformierten Keimzentren und Plasmazellexpansion und lassen sich bereits HE-morphologisch erfassen [3, 11]. Ist die Histologie mit einem CD vereinbar, muss noch die histologische Abgrenzung eines iMCD gegenüber den wichtigsten Differenzialdiagnosen erfolgen (Abb. 6). Schwierigkeiten können sich insbesondere im Rahmen der frühen Krankheitsphase ergeben, wenn regressive Keimzentren nur spärlich vorhanden sind. Verwechslungsgefahr kann durch reaktiv vermehrte Immunoblasten und/oder oligoklonale T‑Zell-Expansionen bestehen, welche auch beim CD vorkommen können und in Bezug auf eine infektiöse Genese oder Lymphome zur Abgrenzung eines iMCD abzuklären sind [3, 16, 17]. Als histomorphologische Differenzialdiagnosen zum iMCD sind insbesondere plasmazellulär ausreifende Lymphome (Ausschluss über Leichtkettenimmunhistochemie und destruierte Lymphknotenarchitektur) sowie HIV-assoziierte Lymphadenopathien aufzuführen, wobei die Abgrenzung insbesondere beim HHV-8-positiven MCD gegenüber verschiedenen KSHV-assoziierten Erkrankungen herausfordernd sein kann (Ausschluss über kaposiforme vaskuläre Transformation und Immunhistochemie; cave: z. B. monotypische λ‑Leichtkettenexpression durch Plasmablasten bei der HHV-8-assoziierten germinotropen lymphoproliferativen Erkrankung). Der Ausschluss von Erkrankungen mit Castleman-ähnlicher Histologie ist auch Teil der internationalen Konsensuskriterien bei der iMCD-Diagnose (Tab. 2; [3]). Vor der Exstirpation und histologischen Aufarbeitung eines Lymphknotens ist auch die CT-basierte Bildgebung als sinnvolles Screeninginstrument im Hals‑, Thorax- und Abdomenbereich zu beachten (ggf. PET-CT). Die Lymphknotenentnahme sollte spätestens bei therapierefraktärer Lymphadenopathie obligatorisch erfolgen. Sofern früher möglich, sollte die Entnahme idealerweise vor Einleitung einer immunmodulatorischen Therapie, wie z. B. einer Steroidgabe, erfolgen. Insbesondere Glukokortikoide wirken breit suppressiv auf Zellen des angeborenen und adaptiven Immunsystems [18] und sind gemäß den aktuellen Leitlinien (Stand Mai 2023) fester Bestandteil zahlreicher Lymphomtherapien inklusive Plasmazellneoplasien. Es ist daher zu vermuten, dass Glukokortikoide hemmend auf aktive, proliferierende Komponenten des Morbus Castleman wie Plasmazellen und Keimzentren wirken, v. a. im frühen Stadium auf die Keimzentrumsreaktion via Inhibition T‑follikulärer Helferzellen [19]. Ob dies wiederum vermehrt zu regressiven Keimzentren führt, wurde bislang nicht systematisch untersucht. Bereits regressive Keimzentren dürften aufgrund ihrer Hypozellularität aber unter Steroidgabe eher persistieren. Vom histopathologischen Standpunkt her ist nicht nur eine attenuierte Grunderkrankung schwerer zu diagnostizieren, sondern auch die mögliche zusätzliche Überlagerung durch Infekte bei erhöhter Vulnerabilität unter Glukokortikoidtherapie schwer abzuschätzen.

**Table 2:** 

**Hauptkriterien (beide erforderlich)**	
Lymphknoten mit typischer Histologie und	
Lymphknoten (Vergrößerung um ≥ 1 cm) an mindestens 2 Stationen	
**Nebenkriterien (mindestens 2/11 Nebenkriterien einschließlich mindestens 1 Laborkriterium)**	
*Labor*	CRP (> 10 mg/l) oder BSG (> 15 mm/h) erhöht
	Anämie (Hb < 12,5 g/dl bei Männern, Hb < 11,5 g/dl bei Frauen)
	Thrombozytopenie (Thrombozyten < 150/μl) oder Thrombozytose (Thrombozyten > 400/μl)
	Hypalbuminämie (Albumin < 3,5 g/dl)
	Nierendysfunktion (eGFR < 60 ml/min/1,73) oder Proteinurie (Gesamteiweiß 150 mg/24 h oder 10 mg/100 ml)
	Polyklonale Hypergammaglobulinämie (> 1700 mg/dl)
*Klinik*	B‑Symptomatik (Nachtschweiß, Fieber, Gewichtsverlust oder Fatigue)
	Splenomegalie und/oder Hepatomegalie
	Ödeme, Anasarka, Aszites, Pleuraergüsse
	„Eruptive“ Hämangiomatosen
	Lymphozytoide interstitielle Pneumonie
**Ausschlusskriterien**	
Infektionsassoziierte Erkrankungen: HHV‑8 (PCR, LANA-1-Färbung per Immunhistochemie), klinische EBV-lymphoproliferative Erkrankungen, Lymphadenopathie durch HIV, CMV, Toxoplasmose, Tuberkulose	
Autoimmun-/autoinflammatorische Erkrankungen (Antikörper allein schließen den iMCD nicht aus): systemischer Lupus erythematodes, rheumatoide Arthritis, Still-Krankheit im Erwachsenenalter, juvenile idiopathische Arthritis, autoimmunes lymphoproliferatives Syndrom	
Maligne bzw. lymphoproliferative Erkrankungen (vorher oder zeitgleich diagnostiziert): Hodgkin- und Non-Hodgkin-Lymphome, multiples Myelom, primär im Lymphknoten lokalisiertes Plasmozytom, FDC-Sarkom, POEMS-Syndrom	

## Differenzialdiagnose von Autoimmunerkrankungen

**Figure 7:**
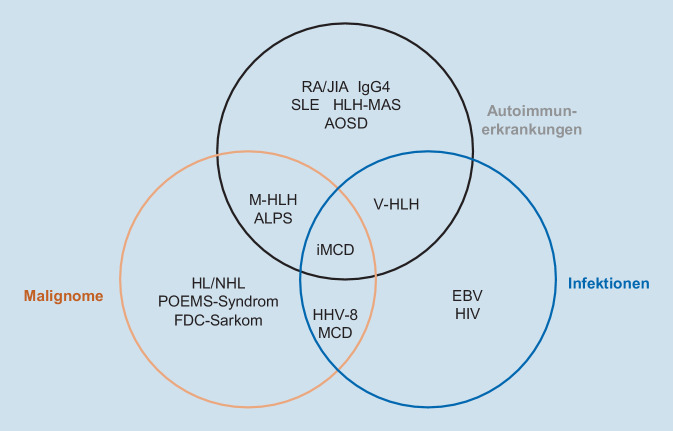

Im Unterschied zum UCD ist das klinische Bild von Patienten mit MCD häufig durch unspezifische Symptome wie Fieber, Nachtschweiß und Gewichtsabnahme (B-Symptome) und ein ausgeprägtes Krankheitsgefühl geprägt, die für eine schwere, hochentzündliche Systemerkrankung sprechen. Das Spektrum der aus klinischer Sicht differenzialdiagnostisch möglichen Autoimmunerkrankungen beim iMCD umfasst den systemischen Lupus erythematodes (SLE), das Sjögren-Syndrom, die rheumatoide Arthritis (RA), die Still-Krankheit im Erwachsenenalter („adult-onset Still disease“ [AOSD]), die juvenile idiopathische Arthritis (JIA), das autoimmune lymphoproliferative Syndrom (ALPS), die IgG4-assoziierten Erkrankungen („IgG4-related diseases“ [IgG4-RD]) sowie Hyperinflammationssyndrome wie das Makrophagenaktivierungssyndrom (MAS-HLH), hämophagozytische Lymphohistiozytose (HLH), das VEXAS-Syndrom (VEXAS = „vacuoles, E1 enzyme, X‑linked, autoinflammatory, somatic“) sowie eine fulminante Sarkoidose. Weiterhin müssen Erkrankungen maligner Genese, Infektionserkrankungen (akute Infektionen mit dem Epstein-Barr-Virus [EBV] oder HIV) sowie überlappende Syndrome (Overlap-Syndrome) differenzialdiagnostisch in Betracht gezogen werden (Abb. 7; [3]). Zudem hat sich das Spektrum der differenzialdiagnostisch relevanten Infektionserkrankungen im Zuge der COVID-19-Pandemie um SARS-CoV‑2 erweitert (SARS-CoV-2-induzierte Zytokindysregulation).

## Systemischer Lupus, Sjögren-Syndrom

Wie die eingangs vorgestellte Kasuistik veranschaulicht, kann nach Ausschluss von Infektionen und Tumorerkrankungen insbesondere bei länger andauerndem Verlauf eine klinische Befundkonstellation resultieren, die an eine Kollagenose denken lässt. Bei etwa 30 % der Patienten mit iMCD wurden Autoantikörper wie antinukleäre Antikörper (ANA) nachgewiesen, da diese im Zuge der polyklonalen Hypergammaglobulinämie ansteigen können und das Vorliegen eines iMCD nicht ausschließen [3]. Umgekehrt können bis zu 59 % der Patienten mit SLE eine Lymphadenopathie aufweisen [20]. Bestehen weiterhin noch ein Pleuraerguss, eine Anämie und eine Thrombozytopenie, erscheint die Diagnose eines SLE naheliegend [21], zumal als klinische Kriterien beim CD lediglich Hautmanifestationen wie eruptive Hämangiomatosen oder violette Papeln atypisch für eine Kollagenose sind. Die Hinterfragung einer vordiagnostizierten Kollagenose bzw. histologische Re-Evaluation ist v. a. dann angezeigt, wenn der klinische und serologische Verlauf nicht zur SLE-Diagnose passen, der Patient männlichen Geschlechts bzw. bei Erstmanifestation im fortgeschrittenen Alter ist oder auf die bisherige Therapie nicht angesprochen hat. Ein SLE ist hingegen wahrscheinlicher bei typischen oder histologisch bestätigten Haut- und Schleimhautmanifestationen, neuropsychiatrischer Beteiligung, Arthritis, Glomerulonephritis, Nachweis spezifischer Antikörper (wie homogene ANA und korrespondierende dsDNS- oder Sm-Antikörper) und Komplementverbrauch.

Die differenzialdiagnostische Abgrenzung zum Sjögren-Syndrom (SS) gelingt bei langjährig bekannter Sicca-Symptomatik, der Histologie sowie durch die Sonographie der großen Speicheldrüsen sowie dem Nachweis der typischen Autoantikörper gegen SSA(Ro)/SSB(La). Kasuistisch wurden Fälle von iMCD berichtet, deren Diagnose durch ein begleitendes SS verkompliziert wurde [22].

Beide Kollagenosen gehen – im Gegensatz zu CD – in der Regel mit vergleichsweise niedrigem/normalem CRP einher.

## IgG4-assoziierte Erkrankung

Eine weitere wichtige Differenzialdiagnose in der rheumatologischen Praxis stellt ein iMCD bei der IgG4-assoziierten Erkrankung („IgG4-related disease“ [IgG4-RD]) dar. Die Unterscheidung ist auch deshalb von Bedeutung, da sich die Therapie der beiden Entitäten erheblich voneinander unterscheidet. Die Beteiligung von Lymphknoten ist bei iMCD obligat, wird aber auch bei den IgG4-RD beobachtet – mit der potenziellen Ausbildung großer abdomineller Raumforderungen bei beiden Erkrankungen. Auch eine Infiltration des Lungen- oder Nierenparenchyms [23, 24] wurde sowohl beim iMCD als auch bei den IgG4-RD beschrieben, wobei anhand der Befunde bildgebender Verfahren alleine eine Unterscheidung nicht möglich ist. Beide Erkrankungen können mit einer Hypergammaglobulinämie und einer Erhöhung der IgG4-Konzentration im Serum sowie in der Gewebebiopsie einhergehen. Bei Patienten mit IgG4-RD wurde eine im Schnitt signifikant höhere IgG4/IgG-Ratio beobachtet als bei Patienten mit iMCD. Neben erhöhten IgG- und IgM-Spiegeln wurden persistierend erhöhte CRP-Spiegel als serologische Ausschlusskriterien für IgG4-RD vorgeschlagen [25].

Bei fehlendem Lymphknotenbefall lässt sich ein iMCD praktisch ausschließen, und auch die Beteiligung von Orbita, Speicheldrüsen und Pankreas spricht gegen diese Diagnose. Dagegen ist eine Splenomegalie atypisch für eine IgG4-RD. Beim iMCD finden sich häufig erhöhte IL-6-Konzentrationen [26], aber auch höhere CRP-, IgA- und IgM-Spiegel sowie häufiger Fieber. Dagegen beruht die Immunpathogenese bei der IgG4-RD auf einer IL-4-getriebenen Aktivierung von Th2-Zellen, weswegen hier auch häufiger eine atopische Diathese beobachtet wird (Tab. 3; [27]). Histologisch charakteristisch für die IgG4-RD im Lymphknoten ist eine Expansion der Keimzentren und in parenchymatösen Organen die wirbel- oder radspeichenartige Fibrose. Prinzipiell ist auch hier im Falle eines schlechten Therapieansprechens auf Glukokortikoide [28] die grundsätzliche Überprüfung der IgG4-RD-Diagnose angezeigt und zu hinterfragen, ob die Symptomenkonstellation möglicherweise mit einer anderen immunologischen Systemerkrankung wie einem iMCD besser vereinbar wäre.

**Table 3:** 

Charakteristika	IgG4-RD	iMCD
*Klinisch*		
Atopie in der Vorgeschichte	Häufig	Normal
Beteiligung exokriner Drüsen	Häufig	Selten
LK-Beteiligung	Gelegentlich	Immer
*Biomarker*		
Hämoglobin	Normal	Niedrig
Thrombozyten	Normal	Hoch
Albumin	Normal	Niedrig
IgG4:IgG-Ratio^a^	Hoch	Normal
IgA	Normal	Hoch
IgM	Normal	Hoch
IL‑6	Normal	Hoch
*Histologie*		
Vergrößerte Keimzentren	Häufig	Gelegentlich
Vermehrung reifer Plasmazellen	Selten	Häufig
Hämosiderinablagerung	Selten	Häufig
IgA^+^-Zellen	Selten	Ausgeprägt

^a^Als pathologisch gilt bei den IgG4-RD eine IgG4:IgG-Ratio > 40 % [25]

## Morbus Still des Erwachsenen

Stark erhöhte Akute-Phase-Proteine, Leukozytose, Fieber, Schluckbeschwerden und vergrößerte Halslymphknoten lassen auch an einen Morbus Still des Erwachsenen (AOSD) denken. Charakteristisch für AOSD sind bizyklische Fieberschübe am Morgen und am Abend sowie ein flüchtiges, kleinfleckig konfluierendes Exanthem insbesondere während eines Fieberschubs. Für die Diagnose des AOSD können die Yamaguchi-Kriterien herangezogen werden [1], die sich auch ohne das Still-Exanthem erfüllen lassen (Still-Syndrom). Vor ihrer Anwendung müssen allerdings Infektionen, hämatologische Erkrankungen und andere rheumatologische Erkrankungen ausgeschlossen werden. Insbesondere bei Abwesenheit des (charakteristischen) Still-Exanthems sollten weitere Differenzialdiagnosen in Betracht gezogen werden. Laborchemisch erwarten wir beim AOSD im akuten Schub eine ausgeprägte Hyperferritinämie, welche bei CD so nicht zu finden ist. Bei anhaltenden Schwellungen der Halslymphknoten über 4 Wochen oder der signifikanten Vergrößerung einzelner oder mehrerer Lymphknoten über 1 cm empfiehlt sich die möglichst komplette diagnostische Entfernung eines gut zugänglichen Lymphknotens.

## AOSD-ähnliche Erkrankungen

Histopathologisch kann die Abgrenzung gegenüber infektionsbedingten Erkrankungen herausfordernd sein: Gerade bei jüngeren Patienten sollte aber auch an die Möglichkeit einer infektiösen Mononukleose (Epstein-Barr-Virus-Erstinfektion) gedacht werden, wobei die lymphozytären Reizformen in den manuellen Ausstrichen des peripheren Blutes, auch Virozyten genannt, richtungsweisend sind. Eine Bestätigung kann mittels EBV-DNS-Nachweis erfolgen. Dabei kann eine chronisch aktive EBV-Infektion auf einen primären Immundefekt (PIK3C-, CD27- oder CD70-Defizienz, Mutationen in SH2D1A oder XIAP) hinweisen [25, 26, 29, 30]. Bei älteren männlichen Patienten ohne Leukozytose, aber mit makrozytärer Anämie und weiteren Hinweisen auf eine dysplastische Störung der Blutbildung (myelodysplastisches Syndrom vom Typ chronische myelomonozytäre Leukämie) sollte auch das VEXAS-Syndrom ausgeschlossen werden. Hierbei handelt es sich um eine heterogene inflammatorische Erkrankung, die durch erworbene somatische Mutation des UBA1-Gens auf dem X-Chromosom verursacht wird und meist mit MDS-ähnlichen Blutbildveränderungen einhergeht. Phänotypisch kann ein VEXAS-Syndrom an eine Pseudo-AOSD-Erkrankung erinnern [31]. Hier können ebenfalls Hauterscheinungen auftreten, die jedoch nicht mit einem Still-Exanthem zu verwechseln sind, daneben auch Manifestationen wie Polychondritis oder Vaskulitis [23]. Darüber hinaus können paraneoplastische Syndrome eine AOSD-ähnliche Erkrankung vortäuschen [32].

## Makrophagenaktivierungssyndrom und HLH

Bei einem Teil der Patienten mit AOSD und bei anderen hochentzündlichen Systemerkrankungen kann ein sekundäres Makrophagenaktivierungssyndrom (MAS) beobachtet werden. Ein primäres MAS kann in der frühen Kindheit auftreten (bezeichnet als hämophagozytische Lymphohistiozytose [HLH]), wobei neben den Fieberschüben und Lymphknotenschwellungen hohe Ferritin‑, AST- und LDH-Spiegel sowie eine zunehmende uni-, bi- oder trilineare Zytopenie möglich sind. Die Hämophagozytose kann in 50 % der Ausstriche von Knochenmarkaspiraten nachgewiesen werden. Sensitivität und Spezifität der Diagnose MAS-HLH können mit dem HScore bestimmt werden [33].

## Therapie des iMCD

Da ein iMCD potenziell lebensbedrohlich ist, ist eine unverzügliche Therapieeinleitung angezeigt. Der monoklonale Anti-IL-6-Antikörper Siltuximab wird basierend auf Daten aus einer randomisierten klinischen Studie von internationalen Leitlinien als bevorzugte Erstlinientherapie empfohlen und ist der einzige von EMA und FDA zugelassene Wirkstoff für den iMCD [5, 34]. Die Zulassung basierte auf einer randomisierten Studie, in der 79 Patienten mit iMCD entweder alle 3 Wochen mit Siltuximab oder Placebo behandelt wurden [34]. Primärer Endpunkt war ein über mindestens 18 Wochen anhaltendes Ansprechen, gemessen durch eine Reduktion der Tumorgröße und Verbesserung der klinischen Symptomatik. Während in der Placebogruppe kein Ansprechen zu beobachten war, lagen das dauerhafte Tumor- und Symptomansprechen in der Verumgruppe bei 34 %. Ein dauerhaftes symptomatisches Ansprechen konnte im Siltuximab-Arm bei 57 % beobachtet werden [34]. In der Regel sprechen die Patienten nach 3 bis 4 Gaben an (Labor, Klinik), ein Rückgang der Lymphadenopathie wird erfahrungsgemäß später beobachtet (initiale Kontrollen alle 3 Monate mittels Bildgebung). In den meisten Fällen muss eine IL-6-inibitorische Therapie dauerhaft fortgesetzt werden [5, 35]. Zur Verträglichkeit und Wirksamkeit von Siltuximab sind inzwischen Langzeitdaten über 6 Jahre verfügbar [32]. Insbesondere zu Beginn sollte eine Kombination mit Kortikosteroiden erfolgen (Prednison 1–2 mg/kgKG), welche über 4 bis 6 Wochen ausgeschlichen werden [35]. Steht Siltuximab nicht zur Verfügung, kann Tocilizumab eine alternative Therapieoption sein [36, 37]. Bei Patienten mit einem milden Schweregrad und geringer klinischer Symptomatik kann zunächst auch ein Therapieversuch mit Rituximab plus Steroiden erwogen werden [35]. In der internationalen Leitlinie wird, abhängig vom Krankheitsbild, für die Zweitlinie der Einsatz von Rituximab in Kombination mit Immunmodulatoren oder einer Polychemotherapie beschrieben [36]. Entsprechend zügig sollte im Falle eines Therapieversagens bzw. bei hochaktivem Krankheitsbild gemeinsam mit den Hämatoonkologen auch die Entscheidung zu einer potenziellen Polychemotherapie (CHOP+/−R, VDTPACE, CVAD+/−R) getroffen werden. Bei Patienten, die auf eine Anti-IL-6-Therapie nicht ansprechen oder sich in der Rezidivsituation befinden, basieren die Therapieempfehlungen meist auf einer Evidenz aus Fallserien oder Einzelfallberichten [38]. In den letzten Jahren konnte eine Aktivierung der mTOR-Kinase und des JAK/STAT-Signalweges beim iMCD gezeigt werden, sodass künftig auch diese Angriffsziele einen therapeutischen Ansatzpunkt bieten könnten [39]. Zur Beurteilung des Therapieansprechens bzw. zum Monitoring der Krankheitsprogression können neben laborchemischen Parametern auch Bildgebungsverfahren (z. B. FDG-PET/CT) herangezogen werden [40].

## Fazit

- Im Unterschied zur unizentrischen Castleman-Krankheit (UCD) handelt es sich bei der idiopathischen multizentrischen CD (iMCD) nicht um eine lokalisierte, operativ behandelbare Lymphknotenhyperplasie: Beim iMCD liegt eine schwerwiegende, potenziell letal verlaufende, systemisch progrediente Erkrankung vor.
- Die Seltenheit der Erkrankung bedingt die eingeschränkte Datenlage zur Häufigkeit des iMCD, doch dürften Inzidenz und Prävalenz prinzipiell eher unterschätzt sein: Durch die heterogene Klinik, Verläufe und Überlappungen mit malignen, infektiös oder autoimmun bedingten Erkrankungen besteht eine hohe Wahrscheinlichkeit für Fehldiagnosen bzw. unerkannte Fälle.
- Allein das Spektrum der differenzialdiagnostisch wichtigen immunmediierten Erkrankungen reicht von SLE/SS, RA/JIA, Morbus Still des Erwachsenen über AOSD-ähnliche Erkrankungen, ALPS, IgG4-assoziierte Erkrankungen bis hin zu HLH und VEXAS-Syndrom.
- Aus Pathologensicht ist es entscheidend, frühzeitig an die Differenzialdiagnose einer iMCD zu denken. Zur optimalen histologischen Beurteilung ist ein vollständiger Lymphknoten erforderlich, eine Punktion ist nicht ausreichend.
- Beim iMCD stellt Siltuximab eine zugelassene medikamentöse Therapie dar. Das Therapieprinzip besteht in der Hemmung des IL-6-Signalwegs. Alternativ könnten andere IL-6-Inhibitoren oder in milden Fällen Rituximab als individueller Heilversuch eingesetzt werden.
